# Dental students’ perspectives on three intraoral scanners and CAD/CAM systems before and after a pre-clinical elective course in digital dentistry

**DOI:** 10.4317/jced.59923

**Published:** 2022-10-01

**Authors:** Panagiotis Zoidis, Naeem Motlagh, Sara Tarte, Connor Vaughan, Lynn Phu, Laura Vandewater, Nura Abujbara, Glenda De La Paz, Muhammad Al-Shafadi, Dayane Oliveira, Mateus-Garcia Rocha

**Affiliations:** 1 Clinical Associate Professor and Dean, Clinical Affairs and Quality Assurance, Department of Restorative Dental Sciences, College of Dentistry, University of Florida, Gainesville, FL, USA; 2 DMD Student, College of Dentistry, University of Florida, Gainesville, FL, USA; 3 Resident, Division of Prosthodontics, Department of Restorative Dental Sciences, College of Dentistry, University of Florida, Gainesville, FL, USA; 4 Clinical Assistant Professor, Center for Dental Biomaterials, Department of Restorative Dental Sciences, College of Dentistry, University of Florida, Gainesville, FL, USA

## Abstract

**Background:**

Intraoral scanners (IOS) are gaining interest in Dentistry for their ability to capture digital impressions of the oral cavity. These digital impressions facilitate the fabrication of indirect restorations using CAD/CAM technology. This study aimed to describe an elective course given to predoctoral dental students on the topic of Digital Dentistry and assess their learning outcomes and system preferences.

**Material and Methods:**

Three IOS were evaluated by eight students enrolled in a Digital Dentistry elective course. These systems included Emerald S (Planmeca), Cerec Omnicam (Denstply Sirona), and True Definition (3M/Midmark). After a literature review and a hands-on session were completed for each system, the students provided their perspectives on various factors such as ease of use, organization, and user-friendliness in a qualitative narrative of each system and quantitatively through a six-items survey.

**Results:**

Survey data suggests that the student cohort showed higher levels of previous familiarity, user preference, and clinical confidence in the Cerec and Planmeca systems as opposed to the True Definition system. Qualitatively, the students felt CEREC was the more educationally useful system to learn and presented with more ease of use, functionality, and efficacy than the other two systems.

**Conclusions:**

While each system proved to have its unique benefits and drawbacks, students’ attitudes towards the Planmeca and Cerec systems were generally positive, while True Definition’s evaluation was limited. Students appreciated their experiences throughout this elective, familiarizing themselves with various digital systems.

** Key words:**Digital Dentistry, Intraoral Scanners, CAD/CAM, Dental Education, Learning Curve.

## Introduction

Intraoral scanners (IOS) use direct optical imaging to create a digital file (1,2). These devices are frequently used to capture a 3D image of a patient’s dentition and surrounding soft tissue to fabricate a prosthetic reconstruction (1,3). Intraoral scanning is growing in popularity with practitioners and academics due to its advantages over conventional impression methods (3). Likewise, chairside design and manufacturing systems have increased efficiency and outcomes for practitioners and patients.

Education in Digital Dentistry is now becoming a widely valued facet of many dental training institutions worldwide, and increasingly, students are craving this sort of education to propel their careers into the new age of dentistry (4). However, faculty members and course directors should be aware that not all systems present user-friendly interfaces, which might impair the student’s digital dentistry learning curve. Although the younger student generations are eager to implement technology in their pre-clinical and clinical learning workflow, struggling with digital systems can create frustration and a lack of interest from students and faculty.

Understanding the students’ perspective on training in digital dentistry is fundamental for institutions to select the ideal IOS that can fulfill the requirements of both pre-clinical and clinical courses (5). Unfortunately, there has been no detailed investigation of the dental students’ perspectives before and after training in digital dentistry using different IOS. Thus, the purpose of this study was to document a predoctoral elective course to introduce students to three computer-aided design/computer-aided manufacturing (CAD/CAM) systems and to provide the perspective of dental students enrolled in the course after studying and using the three IOS and CAD/CAM systems. The null hypothesis was that there would be no difference in the preferences of dental students for a particular IOS and CAD/CAM system.

## Material and Methods

Three IOS were tested in this study: Emerald S (Planmeca, Helsinki, Finland), Cerec Omnicam (Dentsply Sirona, Charlotte, NC, USA), True Definition Scanner (3M, Saint Paul, MN, USA; currently owned by Midmark, Dayton, OH, USA). This study received IRB approval (IRB202102632). Eight dental students voluntarily enrolled in a Digital Dentistry Elective course. The study was divided into two parts. In the first part, three trained professor experts in the digital systems Emerald S (Planmeca), Cerec Omnicam (Dentsply Sirona), and True Definition (3M) provided guided literature reviews on all subjects. The students participated in hands-on training with the same experts after their literature review and discussion. The students were trained by faculty in groups of four and thoroughly practiced the scanning and design for all systems and the fabrication of a three-unit fixed dental prosthesis. After these practical sessions, the students then had the option to fill out a six-item questionnaire gauging their opinions of each of the three systems and their perspectives on the elective course in Digital Dentistry.

The questions on the survey are listed as follows: Q1 – “What was your level of experience using the intraoral scanners before the course?” Q2 – “How do you rate the literature review’s influence on the improvement of your knowledge about intraoral scanners?” Q3 – “How do you rate the user-friendliness of the intraoral scanners?” Q4 – “What is your level of confidence in using the intraoral scanners in the DMD clinic under Faculty supervision?” Q5 – “What is your level of confidence in using the intraoral scanners after your graduation?” Q6 – “How do you rate your understanding of intraoral scanners after the elective course?”. All questions were multiple-choice, and the students could answer the questions with the following options: bad, poor, fair, good, or excellent. The survey was sent through an automated platform (Qualtrics, Provo, UT, USA). No personal information from the students was collected. A Kruskal-Wallis test was used to compare the differences between the systems using a level of significance α = 0.05 and β=0.2.

## Results

**Figure 1:**
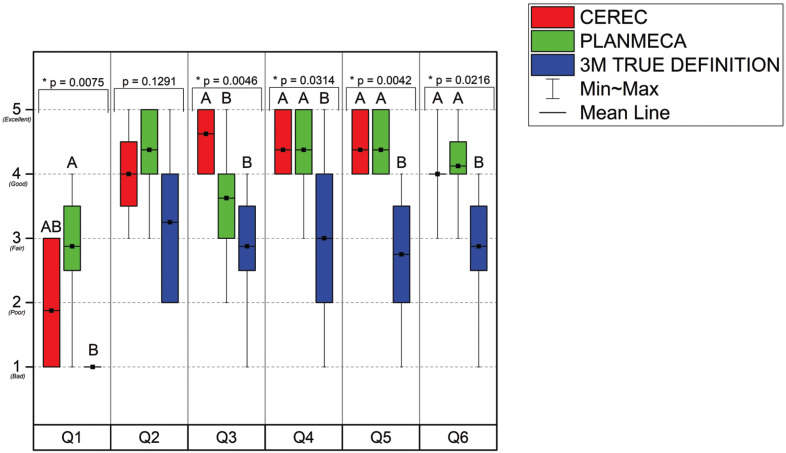
Box-and-whisker plot representation of the six-questions survey.

All students answered the survey at the end of the course to determine learning outcomes. As shown in this box-and-whisker plot representation of the data (Fig. 1), the students reported that they had a better clinical and literacy background in the Emerald S (Planmeca) and Cerec Omnicam (Dentsply Sirona) systems.

For Q1 (“What was your level of experience using the intraoral scanners before the course?”), the students reported that they had a fair to poor experience using the Emerald S (Planmeca); however, no differences in the previous experiences were found between Emerald S (Planmeca) and Cerec Omnicam (Dentsply Sirona). All students reported a previous bad experience with the True Definition Scanner, but no differences were found between True Definition (3M) and Cerec Omnicam (Dentsply Sirona).

For Q2 (“How do you rate the literature review’s influence on the improvement of your knowledge about intraoral scanners?”), the students reported that the literature review improved their knowledge in a fair to excellent range; however, no differences between the systems and the literature review showed efficiency regardless of the IOS. Although there were no significant differences in Q2, the students provided great qualitative feedback about the systems. Key comments were included in this study discussion to demonstrate the value of the literature review on the student’s understanding.

For Q3 (“How do you rate the user-friendliness of the intraoral scanners?”), the students reported that Cerec Omnicam (Dentsply Sirona) is more user-friendly than Emerald S (Planmeca) and True Definition (3M).

For Q4 (“What is your level of confidence in using the intraoral scanners in the DMD clinic under Faculty supervision?”), the students reported a good to an excellent level of confidence for the Cerec Omnicam (Dentsply Sirona) and Emerald S (Planmeca) system under faculty supervision; however, the True Definition (3M) demonstrated a low level of confidence according to the dental students. The same pattern was found for Q5 (“What is your level of confidence in using the intraoral scanners after your graduation?”), showing that the Cerec Omnicam (Dentsply Sirona) and Emerald S (Planmeca) systems provide confidence to the students to work independently.

For Q6 (“How do you rate your understanding of intraoral scanners after the elective course?”), the students reported a good understanding of the Cerec Omnicam (Dentsply Sirona) and Emerald S (Planmeca) system after the elective course; however, even after literature review and hands-on practice, the True Definition (3M) was reported to be fair according to the students’ conceptions.

## Discussion

Prior studies have noted the importance of the learning curve on IOS and CAD system (6,7). These studies emphasize the importance of the user-friendliness of the system and the training method. The null hypothesis of this study that there will be no difference in the preferences of dental students for a particular IOS and CAD/CAM system was rejected. Overall, students’ perspectives corroborate with practitioners that the IOS and CAD/CAM can significantly impact the adoption of digital dentistry workflow (5,8,9).

The students started with the Emerald S (Planmeca). The reading assignments from this first part of the course also included articles to give the students a preliminary understanding of the history and mechanics of digital dentistry. One such article (10) discussed the development of CAD/CAM dental systems dating back to 1971. It then dove into various applications of these systems throughout the years, their pros and cons, and their popularities. The students found this article and other similar ones to be very insightful into the background of these modern systems. They provided a conceptual framework upon which more contemporary findings could be added.

The students then studied a variety of other articles, a few of which will be elaborated upon here for their significance in the course. Hamil *et al*. (9) discussed a student group’s opinion on an educational application of the Planmeca-E4D platform, Compare 2.0. This platform allows students to scan their pre-clinical tooth preparations and compare them to an ideal preparation for feedback and grading. Since this technology is also utilized at the students’ dental college, it was interesting to read about another group of students’ opinions. The study results showed that most students felt that traditional grading methods were somewhat subjective and biased. Therefore, 89% of students in the study were favorable of the approach and thought it would make them better clinicians. This educational application played a role in making our evaluation of this system more beneficial for use in a teaching institution.

One clinically relevant trial studied the quality of 9 intraoral scanners for complete-arch imaging (11). This article also tested the three systems on which this course was focused. The article highlighted each system’s different modes of data capture, which helped the students reinforce the didactic concepts they learned regarding the scientific principles of optical data acquisition. This article also provided an experimental model method for the students to become familiarized with. Like many other experimental studies, the students reviewed this study was an in-vitro comparison of preparation scans to a control scan using an 8-micron range of accuracy lab scanner. After studying the paper’s experimental design and results, the students came to a few points they drew from the article. To not belabor this article, only these concise points of understanding will be listed here: 1. The E4D and Zfx IntraScan IOSs were found to be inferior to the other IOSs’ complete-arch digital scanning. 2. The data capture principle of Swept-source optical coherence tomography (SS-OCT) and the individual image acquisition mode exhibited inferior trueness. The IOSs that required powder coating showed better trueness. 3. The qualitative features varied among IOSs in terms of polygon shapes, sharp edge reproducibility, and surface smoothness.

Another relevant clinical study examined the causes of failure in clinical Cerec restorations (10). The study examined clinical factors: postoperative sensitivity, restoration fracture, color match, margin adaptation, and clinical longevity to determine sources of failure in Cerec inlays and onlays. The article was a systematic review covering 15 clinical studies from 1986-1997. During this period, there was some evolution in these factors: for earlier studies, post-op sensitivity was more common, but in later studies, it was very limited. The restorations had a 16-year survival rate of 85%. And which color and margins deteriorated in esthetics over time, the ditching seen at the margin was not secondary caries seen at the margins.

Finally, a topic that the students in several articles discussed was the limitations of IOS. These limitations were perhaps demonstrated best in the study regarding scanning six implants in complete-arch prosthesis (12). The study attempted to use ten different scanners for this purpose. Consistently the scanners had problems with the reflectivity of the scanners and with being consistent in cross-arch scanning. While IOS is relatively successful at scanning small areas for single teeth or short bridges, it is just as important for the students to discuss these systems’ weaknesses and their impact in a clinical situation. The students were concerned that the implants’ reflectivity could be overcome by using zinc oxide and scanning bodies that do not have metallic surfaces.

The Emerald S (Planmeca) CAD/CAM system was unique to the course because the students had ample exposure to the system throughout their pre-clinical and clinical curriculum. In their previous Pre-clinical Operative Dentistry, General Dentistry, and Prosthodontic courses, students could prepare and scan tooth preparations on a typodont, then design, mill, and cement a ceramic onlay or crown using Planmeca Emerald and Planmill. For this elective course, the students had a new opportunity to design and mill a three-unit fixed dental prosthesis (FDP). The prep, #18-20, was completed on a typodont, and the students used Planmeca Emerald Scan and the Romexis software to scan and design the bridge. The pros of the Planmeca system were the familiarity with the software, scanning, design, and milling process. Planmeca is available on many computers at the University of Florida College of Dentistry. Plenty of scanners make it possible for all students to gain experience and design their restorations. The Planmeca system allows the design to be sent to several milling units in the college, which is a convenient feature. Overall, the Plameca software was user-friendly, but we would like to point out the possible bias in this affirmation as the students had been previously introduced to this system. The cons of the Planmeca system were that the Emerald scanners used were bulky and delayed reading.

Additionally, during the design of the FDP, margin tracing could be challenging to achieve, and the digital tools of restoration design were initially not very straightforward. Due to time constraints in the course, the students did not get the opportunity to fabricate the restorations using Planmill milling unit. However, students are required to manufacture one indirect restoration in a clinical setting. Therefore, the opinion of the students based on clinical experiences is that the process of fabrication of the restoration was comparable to that of Cerec.

The Cerec Omnicam (Dentsply Sirona) system was used to scan and design an FDP bridge extra-orally in a typodont #18-20 for a hands-on learning session for dental students. The pros of the system are that the students felt that the hands-on exposure to the Cerec Omnicam (Dentsply Sirona) system allowed for a holistic view of the procedure from start to finish, allowing for a beneficial learning experience for dental students. Student-designed FDPs allowed for a better understanding and more control over the design outcome than the traditional, lab-designed workflow. The scanner had a compact camera tip with rounded outer edges making it relatively easy to use, and the total scan time took about ~1.5mins. The design software was user-friendly, intuitive, and well-organized in a tab format, providing various tools for altering the design of the FDP. Familiarizing themselves with the design system took the students only approximately 15mins, assuming a level of familiarity with similar design systems.

According to the students’ perspectives, the cons of the experience with the Cerec Omnicam (Dentsply Sirona) system was that the system is generally more expensive than other common CAD/CAM and IOS systems in the market. The students viewed this as a limiting factor for using this system in academic settings. Additionally, being a closed platform, the Cerec Omnicam (Dentsply Sirona) system was viewed by the students as less flexible than others.

For the students’ experience with the 3M True Definition scanner (now owned by Midmark Corp.), titanium dioxide powder was used to spray the FDP preparations on the typodont. The students noticed that the scanner is small, easily manipulated, and user-friendly, allowing a fast-scanning process with accurate results. The 3M system used a blue LED light and video imaging to capture data and create a 3D model during scanning. Some significant cons of the True Definition (3M) system that the students recorded were that the scanner requires a powder coating to be effective, moisture control in the mouth may be a challenge, and no CAD/CAM system is integrated into the clinical side. The scan must be exported to the lab to design and fabricate the bridge.

The students’ overall impressions and comparisons of the three digital systems are as follows. Previous baseline training with the Emerald S (Planmeca) system facilitated the learning curve for all the hands-on components of the course. The students were most familiar with the Emerald S (Planmeca) system and assessed it as student-friendly with clinically accepTable accuracy and precision. Literature analysis reported improved accuracy and precision with the Cerec Omnicam (Dentsply Sirona) system. Students noted similarities in the user experience between the Emerald S (Planmeca) and CEREC systems and that system selection could depend on user preference and training with either software. The True Definition (3M) system was reviewed with the sense that this system is not commonly encountered in current clinical practice. Students found the lack of ability to personally design and mill the restoration to be a significant disadvantage of the True Definition (3M) system.

Among the digital systems reviewed, the students would recommend the Cerec Omnicam (Dentsply Sirona) system for other DMD students to study and practice. The students recognize that finances play a role in practitioners’ selection and availability of digital systems. However, there is practical value in training, with most used in private practice systems. Dental schools should incorporate training with relevant systems as dentistry’s digital workflow becomes more prevalent.

However, they preferred Cerec Omnicam (Dentsply Sirona) due to its user-friendliness. They also felt equally confident in their capacities to use both Cerec Omnicam (Dentsply Sirona) and Emerald S (Planmeca) systems in clinical practice. But it seems that the brevity of the course duration (one academic semester) did not allow for students to reach a maximal understanding (excellent) of the various systems, leaving their understanding levels around the “good” category for Cerec Omnicam (Dentsply Sirona) and Emerald S (Planmeca), and around the “fair” category for True Definition (3M).

## Conclusions

Upon completing a literature review and hands-on sessions with the selected digital scanner systems, the students reflected on their experiences in the course. The Digital Dentistry elective provided unique opportunities to study and interact with aspects and systems of the digital workflow that were not available in the existing curriculum. The small class group size promoted greater student engagement. Students participated in an in-depth discussion regarding clinical applications and were given the opportunity to experience each digital system first-hand. The students appreciated the inclusion of several digital systems for comparison and found the hands-on components to be the most educational and enjoyable.

Further considerations that the students felt may benefit other educational institutions would be having typodonts and stone models available to practice. Along these lines, it may help students to learn more about the lab side of commonly used CAD/CAM systems, further incorporating lab scanners and design software.
